# Is Dysbiotic Gut the Cause of Low Back Pain?

**DOI:** 10.7759/cureus.42496

**Published:** 2023-07-26

**Authors:** Harish V K Ratna, Madhan Jeyaraman, Sankalp Yadav, Naveen Jeyaraman, Arulkumar Nallakumarasamy

**Affiliations:** 1 Orthopaedics, Rathimed Speciality Hospital, Chennai, IND; 2 Orthopaedics, ACS Medical College and Hospital, Dr. MGR Educational and Research Institute, Chennai, IND; 3 Medicine, Shri Madan Lal Khurana Chest Clinic, Moti Nagar, New Delhi, IND

**Keywords:** pain, low backache, lba, gut, dysbiotic

## Abstract

Low back pain (LBP) is the foremost cause of disability that affects the day-to-day activities of millions of people worldwide. The putative trigger of LBP is linked to the gut microbiome (GM) and its dysbiotic environment. With the concept of GM, various disease pathogenesis has been revisited with plausible crosstalks and micromolecular mimicry. In the normal intervertebral disc (IVD), *Firmicutes *and *Actinobacteria* were found in abundance. The blood-disc barrier protects IVD from systemic infection, resists inflammation, and halts the immune surveillance of the inner aspects of IVD. The insights into microbial ecology will broaden our horizons in GM and IVD degeneration in LBP cases. However, an improved understanding of GM and back pain has to be explored in large-scale individuals with varied timescales to validate the above findings. The role of GM (diet, prebiotics, probiotics, and fecal microbiota transplantation) in pain modulation can form novel therapies in cases of LBP.

## Editorial

Low back pain (LBP) is the foremost cause of disability that affects the day-to-day activities of millions of people worldwide. Though there are various causes of LBP, the cause and nature of the inflammation and degeneration of the intervertebral disc (IVD) are not fully understood. The putative trigger of LBP is linked to the gut microbiome (GM) and its dysbiotic environment. GM plays a vital role in the various physiological aspects of the host, viz., metabolism, development, and immunity, and also contributes to host responses like pain and inflammation. Gut dysbiosis is a state characterized by the excessive growth of pathobionts (pathological microorganisms), which has been found to contribute to the pathogenesis of various pathologies like LBP, cancers, and autoimmune diseases. The imbalance between the composition of symbiont and pathobiont decreases the integrity and function of the intestinal barriers, leading to chronic inflammation and inducing pain. Various studies on GM led to the discovery of the intercommunications between the gut and IVD [1,2]. This article provides a relationship between dysbiotic gut and spine disorders.

Normal IVD microbiome

In traditional teaching, it has been taught that the eye, the central nervous system, and IVD are sterile with unique immune privileges and are protected from an uncontrolled inflammatory response. With the development of advanced diagnostics such as 16S rRNA sequencing and shotgun metagenomics, which are culture-independent, researchers have found the presence of microbes in these structures.

With the concept of GM, various disease pathogenesis has been revisited with plausible crosstalks and micromolecular mimicry. In this connotation, Rajasekaran et al. established a normal IVD microbiome with 24 lumbar IVDs. They identified 355 bacterial species, out of which 32 were unique to normal IVD. The species that were evident clinically, viz. *Lactobacillus mucosae* (essential probiotic), *Propionibacterium granulosum* (skin commensal), *Sphingomonas yabuuchiae* (tumour-protective), and *Staphylococcus epidermidis* (oral commensal that prevents pathogen invasion), were seen to be plenty in number in normal IVDs [1].

In the normal IVD, Firmicutes and Actinobacteria were found in abundance, which produce fatty acids with short chains and act as the first barrier of defence in the wall of the gut. These bacteria prevent lymphocyte activation and immunoglobulin production. *Saccharopolyspora*, a bacteria found in normal IVD, is deadly to Gram-positive and Gram-negative bacteria [2]. Table 1 depicts the pathogenic bacteria implicating the inflammatory responses and the pathogenesis of IVD diseases.

**Table 1:** Intervertebral disc bacterial species found in common among gut, skin, and spine IBD: inflammatory bowel disease

Anatomical relationship	Microbe	Clinical significance
Gut and spine	Prevotella corpi	Activates mucosal and systemic T-cell responses which contribute to chronic inflammatory arthritis
	Faecalibacterium prausnitizii	Negatively correlates IBD and colorectal carcinoma
Skin and spine	Acinetobacter johnsonii	May cause infection
	Pseudomonas stuzeri	May cause infective endocarditis
	Pseudomonas nitroreducens	Used in the bioremediation process
	Corynebacterium durum	Plausible role in respiratory tract infections
Gut, skin, and spine	Propionibacterium acnes	Potential role in the pathogenesis of autoimmune diseases, disc degenerations, and Modic changes
	Staphylococcus epidermidis	Skin commensal causing native valve endocarditis

Gut-disc-spine axis

**Figure 1:**
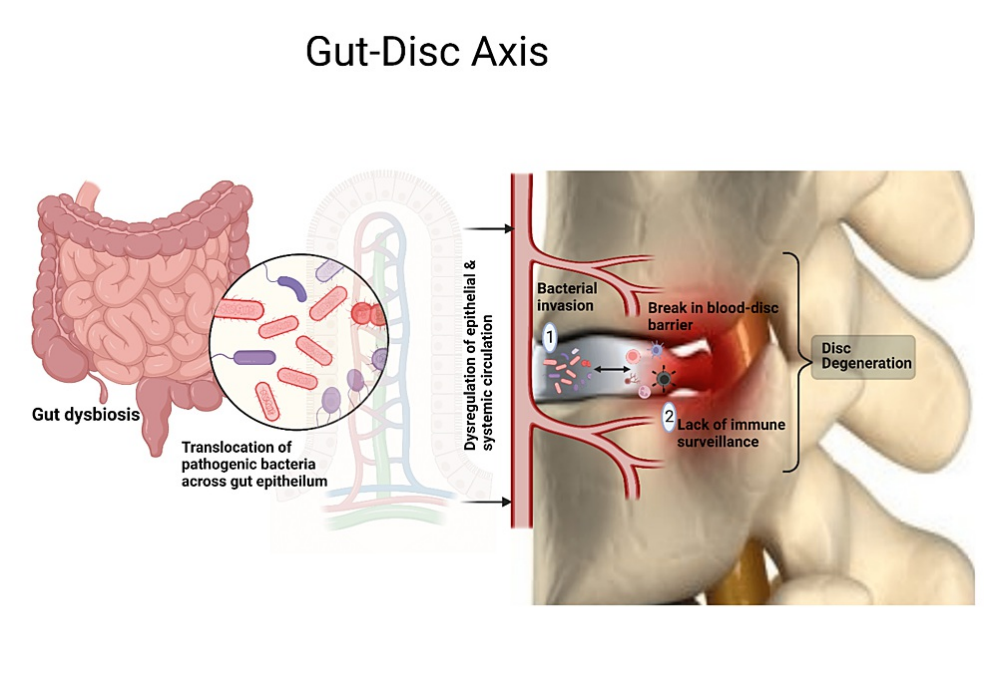
Gut-disc axis Picture courtesy Dr. Madhan Jeyaraman

Gut dysbiosis leads to the abnormal production of several metabolites, signalling molecules, and immune cells that may affect the musculoskeletal system. Li et al. hypothesized that microbes gain access to IVD through a hematogeneous route and postulated three mechanisms of the gut-disc axis that lead to the development of LBP as follows: (a) bacterial translocation across the gut epithelial barrier and into the IVD; (b) systemic and mucosal immune regulation; and (c) balancing the absorption of nutrients and formation of metabolites in the epithelium of the gut and its spread into the IVD, as depicted in Figure 1 [2].

The blood-disc barrier protects IVD from systemic infection, resists inflammation, and halts the immune surveillance of the inner aspects of IVD. Anaerobic bacterial invasion into the IVS causes rapid bacterial multiplication due to the non-availability of immune surveillance and persisting low oxygen levels in the IVD. Further, intestinal inflammation allows the passage of microbes through the epithelial barrier due to enhanced intestinal permeability. This paves the way for the researchers to utilize probiotics in LBP patients.

With the advanced technologies of next-generation sequencing (NGS), the gut microbiome has been sequenced with 500 to nearly 15,000 bacterial populations in the gastrointestinal tract. The bacterial cell population (13 trillion) in the gut outnumbered the number of cells in the body (1 trillion), which play a vital role in various processes such as colonization resistance, immune homeostasis, synthesis of vitamins, and modulation of host metabolisms.

In the literature available, there has been no documentation or consensus regarding the relationship between IVD diseases and the gut microbiome. Recently, Rajasekaran et al. documented about 58 bacteria that are similar between the gut and IVD and 29 bacteria that are similar between the skin and IVD, as shown in Table 1 [1]. There is no direct temporal relationship between the GA and LBP, but there is established evidence regarding the clinical correlation between gastroenteritis and urinary tract infection with LBP [1].

Clinical significance of dysbiotic gut in low back pain

Literature evidence on preclinical models of IVD demonstrated autoimmunity as the plausible cause of inflammation leading to IVD disorders. The immune system is interconnected with the gut microbiome. However, gut dysbiosis leads to mucosal inflammation of the gut, which results in simultaneous changes in the immune system at distant sites. Administration of probiotics changes the equilibrium from a pro-inflammatory [Interleukin (IL)-1, -2, -4, -5, -6, -12, -17A, tumour necrosis factor-alpha (TNF-α), and interferon-gamma (IFN‐γ)] to an anti-inflammatory [IL-10, forkhead box P3 (Foxp-3), and transforming growth factor-beta (TGF-β)] state.

Jensen et al. observed an overall improvement in backache and leg pain at the end of one year with the usage of Lactobacillus rhamnosis GG (6 billion colony-forming units (CFU) in a capsule twice daily for 100 days) compared to the placebo group without any other statistically significant difference in the predefined outcomes in both groups in the cases of LBP with type 1 Modic changes [3].

Wang et al. demonstrated that the use of *Lactobacillus paracasei* S16 in a mouse model of lumbar disc herniation (LDH) attenuates the inflammatory response by producing butyrate, rebuilding GM, and modulating serum metabolomics by facilitating purine metabolism and lowering alanine, aspartate, and glutamate metabolism. Such probiotic administration improved the overall behaviour of the mouse, enhanced cellular proliferation, and decreased apoptosis of the cells. NGS (16s rRNA sequencing) studies exhibited increased levels of *Lachnospiraceae* and *Ruminococcaceae* and decreased levels of Lactobacillaceae bacteria [4].

Dekker Nitert et al. observed a relationship between back pain in obese, healthy individuals and gut dysbiosis. They reported a high abundance of the genera Adlercreutzia, Roseburia, and uncultured Christensenellaceae in patients with backaches and an increased body mass index. Among these three genera, Adlercreutzia exhibited a higher prevalence of back pain across several timescales and follow-ups [5].

Rajasekeran et al. reported the existence of the gut-skin-spine microbiome axis and provided a plausible role for gut dysbiosis in spinal disorders. They insisted on the presence of subclinical infection and inflammation, purporting to be the pathogenesis of IVD degeneration and herniation [1]. The presence of Propionibacterium acnes in IVD is well documented in the literature using 16S NGS, biofilm studies, DNA-based analytic data, and the electron microscopic appearance of IVD cells. Organs and tissues deemed to be sterile were investigated and found to have the existence of a microbiome [1]. With increasing evidence, there is a need for an hour to investigate the microbial population contributing to the evolution of spinal disorders.

Future directives

Rajasekaran et al. provided a paradigm shift and became an eye-opener for all spinal surgeons to explore the human GM in the pathogenesis of IVD degeneration [1]. With its limitations, this study detected the presence of bacteria in normal IVD and pathogenic IVD, which provided the researchers with a platform to explore further phages, viruses, fungi, and parasites within IVD. Furthermore, the insights into microbial ecology will broaden our horizons in GM and IVD degeneration in LBP cases. The inhibition of the inflammatory cascade and amplification cascade in IVD by targeting GM and the IVD microbiome paves a better platform for the management of IVD degeneration and LBP.

To conclude, an improved understanding of GM and LBP has to be explored in large-scale individuals with varied timescales to validate the above findings. The role of GM (diet, prebiotics, probiotics, and faecal microbiota transplantation) in pain modulation can form novel therapies in cases of LBP.
